# Next-generation and further transgenerational effects of bisphenol A on zebrafish reproductive tissues

**DOI:** 10.1016/j.heliyon.2018.e00788

**Published:** 2018-09-15

**Authors:** Afroza Akhter, Mostafizur Rahaman, Ryu-to Suzuki, Yuki Murono, Toshinobu Tokumoto

**Affiliations:** a Integrated Bioscience Section, Graduate School of Science and Technology, National University Corporation Shizuoka University, Oya 836, Suruga-ku, Shizuoka 422-8529, Japan; b Department of Biology, Faculty of Science, National University Corporation Shizuoka University, Oya 836, Suruga-ku, Shizuoka 422-8529, Japan

**Keywords:** Developmental biology, Physiology, Zoology

## Abstract

Next-generation effects and further transgenerational effects of an endocrine disruptor, bisphenol A (BPA), were investigated in zebrafish. The effects of BPA treatment through dietary administration in male and female zebrafish on reproductive factors, such as gonadal activity, fertility, hatching rate and malformation in subsequent generations, were examined through the third filial (F3) generation. BPA treatment of initial generation (F0) not only caused retraction of the ovaries and testes but also lowered the survival rate and increased the rate of malformation of the offspring. Although the overall phenotypes of the surviving first filial (F1) generation offspring of treated fish initially appeared to be normal, we found abnormalities in their reproductive tissues after they matured to adulthood. Although the juveniles were fed a normal diet, the ovaries of 40% of the F1 generation fish remained small and did not develop vitellogenic oocytes. Moreover, sterile male fish appeared at a higher percentage (48%) than control (10%). Adverse transgenerational effects on the fecundity of the second filial (F2) and F3 generation fish were also observed. In each generation, survival rate of embryos were significantly low and abnormal embryos were appeared in offspring from BPA treated ancestral. These results demonstrate that the effects of BPA are transferred to subsequent generations not only through oocytes but also through sperm.

## 1 Introduction

The transgenerational inheritance of the effects of endocrine disruptors has been demonstrated in a series of reports. Abnormalities in offspring have been found in mice and fish. These experimentally induced abnormalities in juveniles were first described in rats (Anway et al., 2005), and the next-generational effect has been studied since this discovery (Daxinger and Whitelaw, 2012). Exposure to endocrine-disrupting chemicals, such as vinclozolin or methoxychlor, in the late embryonic or early postnatal period influences sexual differentiation, gonad formation, and reproductive functions in the first filial (F) generation (F1 generation) (Anway et al., 2005). These effects have been shown to be transferred through the male germ line to nearly all males of all subsequent generations examined (F1 to F3). Epigenetic changes are presumed to be the cause of the transgenerational effects.

In fish, evidence of transgenerational effects has been reported. Paternal inheritance of adverse effects of bisphenol A (BPA), resulting in disruption of cardiogenesis in subsequent generations, has been demonstrated in zebrafish (Lombo et al., 2015). The transgenerational effects of both BPA and 17a-ethinylestradiol exposure on the fertilization capacity and survival of medaka juveniles have been investigated (Bhandari et al., 2015). Recently, deregulation of epigenetic patterns by BPA treatment has been demonstrated (Santangeli et al., 2016). These results suggested that endocrine-disrupting chemicals might have negative effects on populations of fish inhabiting contaminated aquatic environments. As stated above, a substantial number of laboratory animal studies have suggested that the plasticizer BPA may be a reproductive toxicant for humans (Richter et al., 2007; Rochester, 2013). Thus, the actual effects of BPA on organisms should be investigated.

We previously reported an experimentally induced sex change from female to male in adult zebrafish by dietary exposure of 0.2 mg of fadorozole/g of diet (Takatsu et al., 2013). In our parallel experiments, we sought to induce a male to female sex change by administering estradiol (E2) or other compounds that mimic the activity of E2, such as BPA. Surprisingly, our results indicated that all of the offspring of BPA-treated males appeared to be male from their aspect. The oocytes in the ovaries did not develop and ovaries were filled with previtellogenic oocytes. The result suggested that the effects of BPA was inherited to next-generation and caused defect of ovarian development. Recently, transgenerational effect of endocrine disrupting chemicals is receiving attentions as described above. We aimed to confirm feasible effect of BPA on next-generation and also examined transgenerational effect.

In this study, we confirmed the preliminary results of the next-generation effects and further addressed the transgenerational effects of dietary BPA on zebrafish. Our present results suggest that the next-generation effects on zebrafish gonads are induced in both sexes. Furthermore, we demonstrated the transgenerational effects of BPA continue to F3 generation. These results indicate that some factors or changes in the genome are inherited through the germ cells and are passed on to the next generations.

## 2 Materials and methods

### 2.1 Animals

All animal experiments were conducted in accordance with the relevant national and international guidelines and the ‘Act on Welfare and Management of Animals’ (Ministry of Environment of Japan). The transgenic line *TG (β-actin: EGFP)* was established by Hsiao *et al* (Hsiao and Tsai, 2003). Although the cDNA integrated in this strain was constructed for *EGFP* expression driven by the medaka *b-actin* promoter, *EGFP* expression was restricted to the oocytes and gills in adult fish. A *roy* mutant zebrafish, which is deficient in the iridescence exhibited by iridophores, thus resulting in a transparent body, was isolated. We crossed the *roy* and *TG* strains to establish a strain that enables the direct observation of oocytes in living fish. The resulting strain, *TG* (*β-actin: EGFP);roy*, was highly transparent, and its oocytes were easily observed by fluorescence in living fish. We refer this strain as *β-roy* (Akhter et al., 2016; Takatsu et al., 2013). The *TG* zebrafish were bred and maintained at 28.5 °C under a 14-h light/10-h dark cycle (Westerfield, 1995). All zebrafish experiments were carried out with approval from the Institutional Ethics Committee of Shizuoka University, Japan (approved No. 29F-2); the guidelines set by this committee for the usage of animals were strictly followed.

### 2.2 Reagents

The BPA standard for environmental analysis (Cat. No. 025-13541) was obtained from Wako Pure Chemical Industries (Osaka, Japan).

### 2.3 Food preparation and fish treatments

**Fig. 1:**
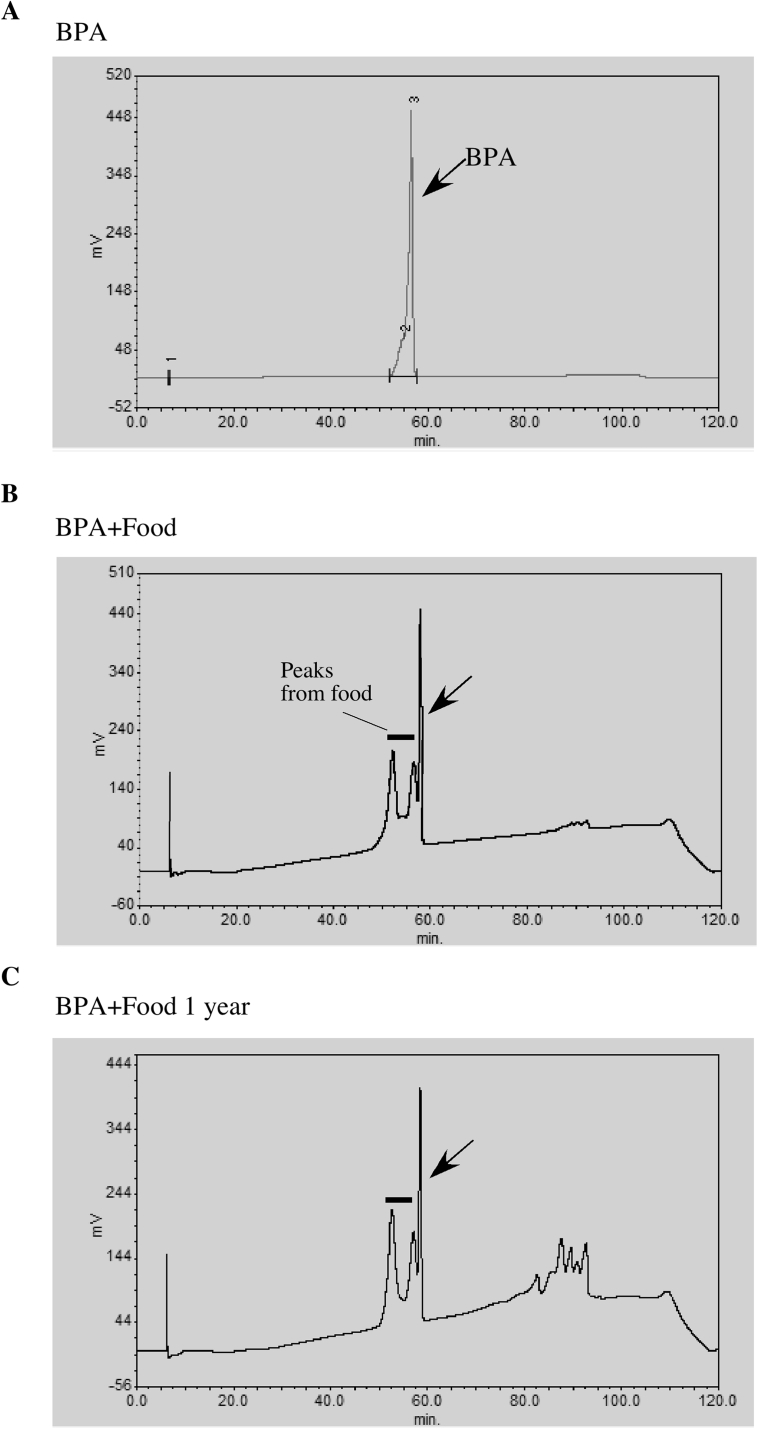
HPLC analysis of BPA mixed in food. BPA was extracted by organic solvent (3 times with hexane and 2 times with diethyl ether: n-hexane = 1:1) from freshly prepared BPA-containing food or stored for one year. Extracted compounds were dissolved in ethanol and analyzed by HPLC. Standard BPA dissolved in ethanol showed a peak at approximately 50% of acetonitrile (A). A corresponding peak was detected in extracts from freshly prepared BPA-containing food (B) and one-year-old food (C).

**Fig. 2:**
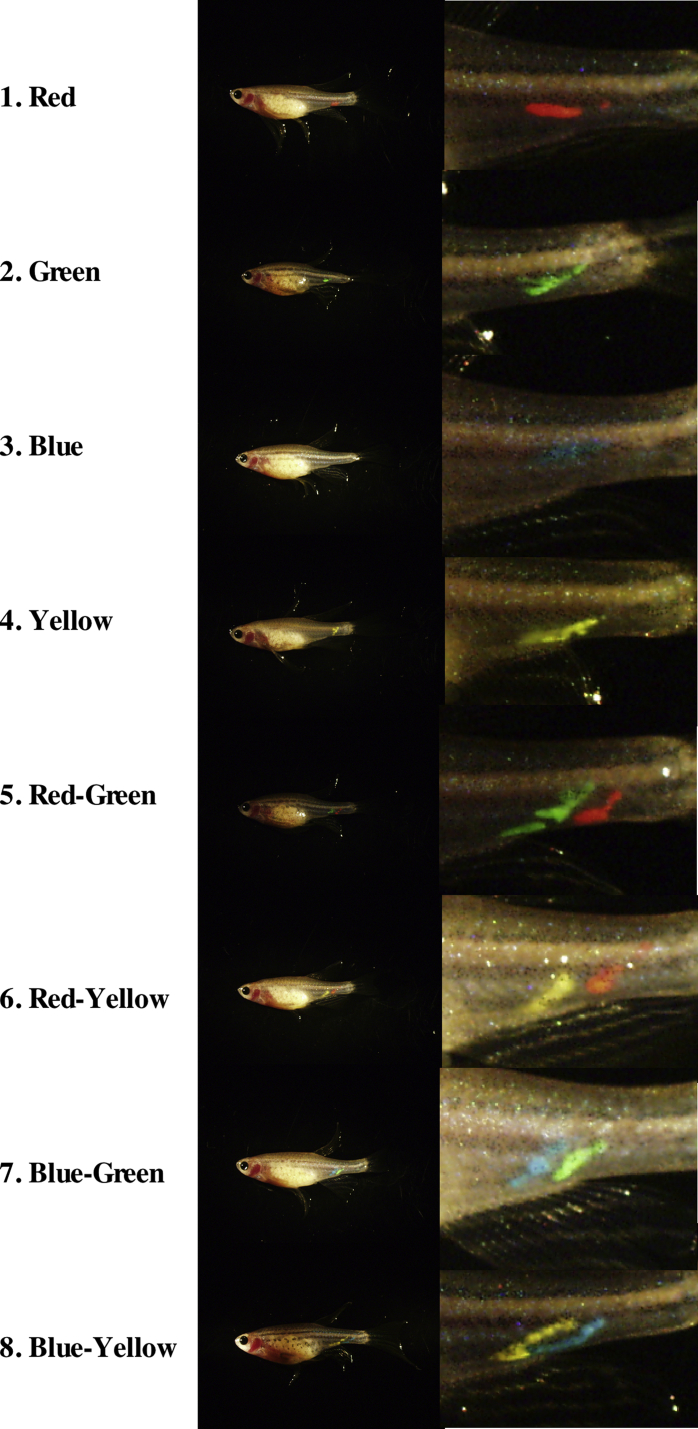
Marking of fish with fluorescent dye. The sexually mature females and males (3-month-old fish of 0.2 ± 0.04 g in body weight) used for the treatments were selected and marked by injection of fluorescent dye (Visible Implant Elastomer Tag, Northwest Marine Technology, NW). Different 8 patterns of color were used to distinguish individuals in a group of 8 fishes. Marked fish were kept in same tank as one experimental group (Fig. 3).

**Fig. 3:**
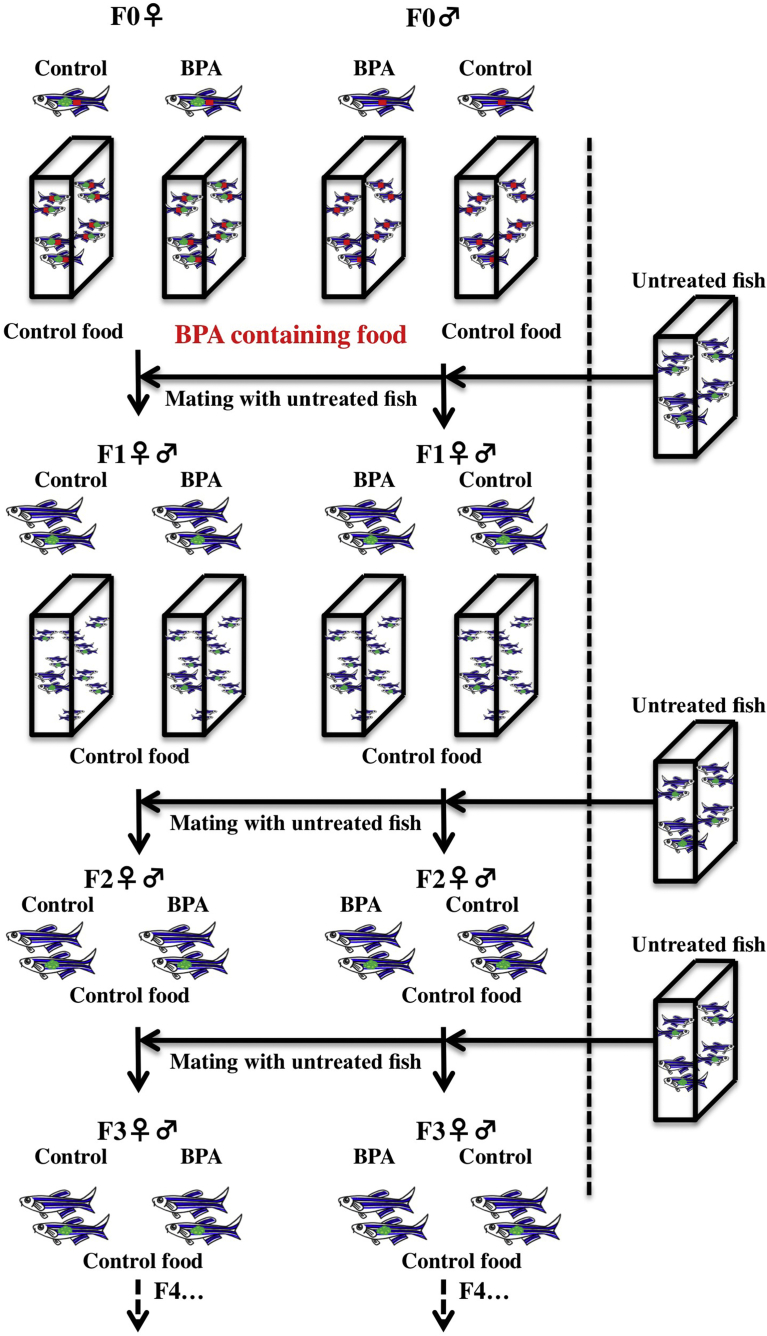
Schematic of zebrafish mating history in this study. Original fish (F0) of each sex were fed BPA-containing food (0.1 mg of BPA/g food) or control food (BPA-non-containing food). After 4 to 6 months of treatment, F0 fish were mated with untreated fish (housed separately from experimental fish) and offspring (the F1 generation) were raised on control food. Fish were separated into four groups, with fish originating from F0 females treated with or without BPA and from F0 males treated with or without BPA as indicated. After 4 to 6 months of culture, F1 fish were mated with untreated fish, and juveniles (the F2 generation) were raised on control food. After 4 to 6 months of culture, F2 fish were mated with untreated fish, and juveniles (the F3 generation) were raised on control food. Serial cultivation was continued through the F2 and F3 generations.

Chemical powder of BPA was mixed with powdered fish food grains (Chroma, Kobe, Japan) by crashing with ceramic mortar and stored at room temperature in a dark box as previously described for sex-change experiment (Akhter et al., 2016; Takatsu et al., 2013). Under these conditions, the BPA mixed in the food was stable for more than one year (Fig. 1). The sexually mature females and males (3-month-old fish of 0.2 ± 0.04 g in body weight) used for the treatments were selected and marked by fluorescent dye (Fig. 2) (Visible Implant Elastomer Tag, Northwest Marine Technology, NW). Marked fish were divided into control and BPA treatment groups consisting of 8 fish each (Fig. 3). Three groups (a total of 24 fish in each treatment) were set. Each group was housed in a separate small aquarium with a filtration system. Aquariums were filled with charcoal-filtered tap water (pH 6.8 to 7.2) and kept at room temperature (25 °C). The fish were fed food containing BPA or not containing BPA twice a day (2 small spoonfuls per feeding corresponding to 13.5 ± 1.3 μg per fish) for 6 months (until the treated fish were 9 months old). The amount of BPA used (0.1 mg/g diet) was determined on the basis of the results of pilot experiments. These fish (the F1 generations) were divided into two groups (generated from an F0 female and from an F0 male) and given normal food (BPA-free food). In each generation, the fertility of the fish was tested by mating the fish with untreated fish after 4 to 6 months of culture. Embryos obtained from paring was cultivated in incubator set at 28.5 °C for 3–4 days. Then, juveniles were released into aquariums. Juveniles were fed paramecium and dried brine shrimp egg powder. Juveniles were kept in larger aquariums until they were 3 months old. Then, experimental fish for use in generating subsequent generations were randomly selected. These fish were fed control food and housed in tanks of the same size as those used for exposure in the F0 generation.

Ovarian morphology was monitored by fluorescence microscopy observations at 1-month intervals during the treatment period (SZX12, Olympus, Japan). After anesthetization with 0.5% tricaine, the ovaries were photographed using a binocular microscope under both bright field and fluorescent lighting conditions. After completion of the fecundity test (6 months), gonadal tissues were excised, and sections were prepared for histological observation.

### 2.4 Fertility check

As described in above, pairings were conducted after 4 (7 months old) to 6 (9 months old) months of exposure treatment more than three times for individual experimental fish. The fish were allowed to mate with untreated females or males. Each mating pair was housed in a plastic aquarium paved with glass beads or in a bottom-meshed aquarium from the evening until the morning of the next day. In the case of a female fertility check, if no egg was obtained, the fertility of the paired untreated male was confirmed by further mating with an ovulation-induced untreated female on the same day. In the case of a male fertility check, if no egg was obtained, the fertility of a paired normal female was confirmed by further mating with another normal male on the same day. Only the results of pairing fish with confirmed fertility were included in the data analysis. In the case, no eggs obtained more than three times from paring with ovulated females, the tested male counted as infertile. Contrary, no eggs obtained more than three times from paring with active males that could not spawn, the tested female counted as infertile.

### 2.5 Analysis of embryos

Eggs were obtained about one hour after turning on the light and kept in a petri dish (15 cm in diameter) from paring tank. The total number of embryos obtained was counted. The numbers of hatched embryos and abnormal embryos were counted after 2–3 days. Morphology of embryos were photographed under a binocular microscope (SZX12, Olympus, Japan). After 3–4 days, juveniles were released into an aquarium.

### 2.6 Histological observation

After a long observation period (6 months of treatment), fish originating from the BPA-treated and control groups (n = 3) were fixed in Bouin's solution for 18 hours and then preserved in 70% ethanol for further analysis. The fixed samples were dehydrated in a graded ethanol series and embedded in paraffin. Eight-micrometer sections were prepared, and standard histological techniques were used to stain the sections with hematoxylin and eosin.

### 2.7 Analysis of sperm

For morphological observation, sperm was collected by ejaculation. Sperm was suspended in Hank's Ringer solution and stained with DAPI. The morphology of sperm was observed by fluorescent microscope (Olympus, BX60, Japan). For counting of number of sperm, testes were obtained from dissected males and minced in 200 μl of Hank's balanced salt solution. Fluorescent nucleus of sperm or spermatozoa-like cells in 1 μl of suspensions were counted (n = 3).

### 2.8 Statistical analysis

All experiments were repeated at least three times. One-way analysis of variance (ANOVA) of the data was analyzed using GraphPad Prism (San Diego, CA, USA). A *P* value of <0.05 was considered statistically significant.

## 3 Results

### 3.1 Effects of BPA on the ovaries and testes of zebrafish (the original parent (F0) generation)

**Fig. 4:**
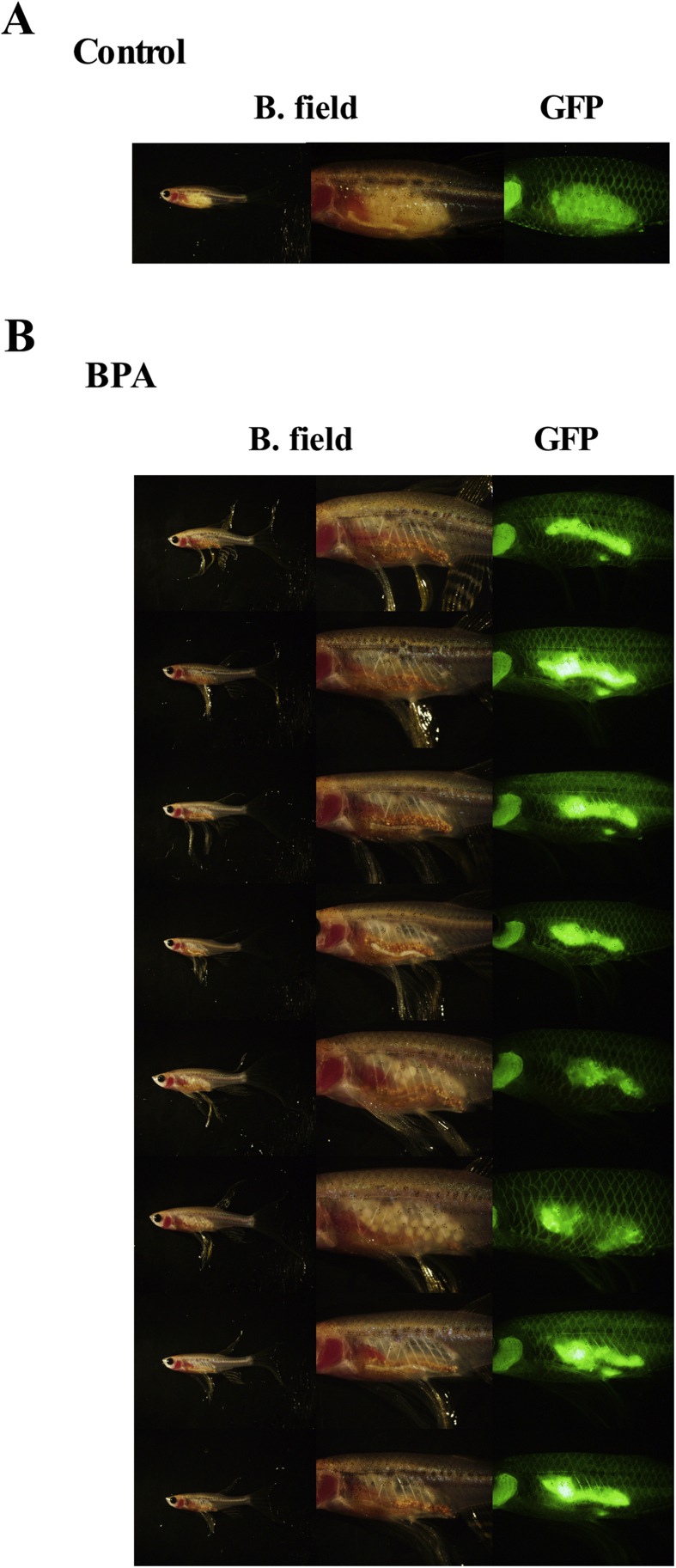
Adverse effects of BPA treatment on ovaries in the F1 generation of zebrafish. (A) Outside view of a normal *β-roy* female. (B) Parental male zebrafish were fed BPA-containing food (10 mg of BPA/g food) for 6 months. Then fish were mated with untreated fish (housed separately from experimental fish) and offspring were raised on control food. Outside view of raised female fish generated from BPA-treated males. Note that all indicated fish are seems like males in bright field but they have fluorescent oocytes in abnormal ovaries. Photographs of whole fish and ovarian tissues under bright field (B. field) and GFP filter views (GFP) are shown.

To monitor the changes in the ovaries and testes of living fish during BPA administration, we used ovarian fluorescent and transparent transgenic zebrafish lines (Fig. 4A) (Akhter et al., 2016; Takatsu et al., 2013). The fluorescence of oocytes in the studied strain was relatively strong in small oocytes at stages 1 and 2. This property was well suited for the observation of undeveloped ovaries. As described in the introduction, the offspring of BPA-treated females showed abnormalities in their gonads (Fig. 4B). The structure of the ovaries was abnormal, and oocytes were not developed. These females were infertile (8 among 8 fish). To confirm this next-generation effect and to further examine the transgenerational effects of BPA, 3 three-month-old fish of each sex were designated as one experimental group and housed in the same aquarium. Similarly, control fish of each sex were separated into groups and fed control food (Fig. 3).

**Fig. 5:**
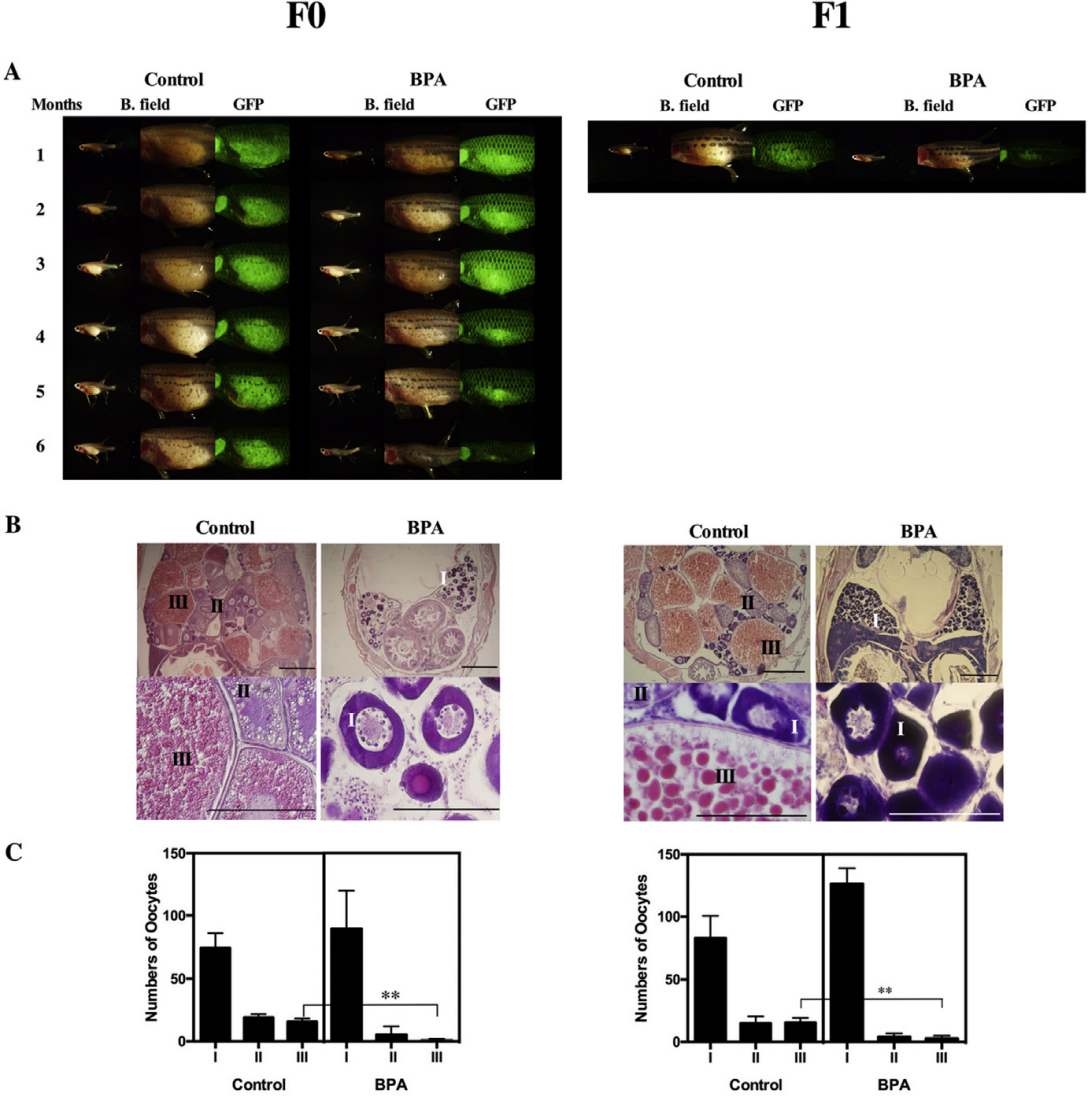
Adverse effects of BPA treatment on ovaries. (A) The morphology of ovaries of F0 females during the treatment was monitored by fluorescence observations performed at 1-month intervals (F0). Photographs of representative F1 females from the control (Control) and BPA-treated (BPA) groups were taken before histological analysis (F1). Photographs of whole fish and ovarian tissues under bright field (B. field) and GFP filter views (GFP) are shown. (B) Sections of control-treated (Control) and BPA-treated (BPA) females. Developmental stages of oocytes are indicated according to Selman et al. as follows: I (early vitellogenic oocytes, perinucleolar oocyte), II (Mid-vitellogenic oocytes) and III (fully grown oocytes) (Selman et al., 1993). The scale bars indicate 50 μm. (C) Number of oocytes in stages I, II or III in sections of control-treated (Control) and BPA-treated (BPA) females were counted in three sections prepared from three different fishes. Vertical lines indicate standard deviation. ** indicate statistically significant differences between the values for the BPA-treated group and the control group at the P < 0.01 level.

**Fig. 6:**
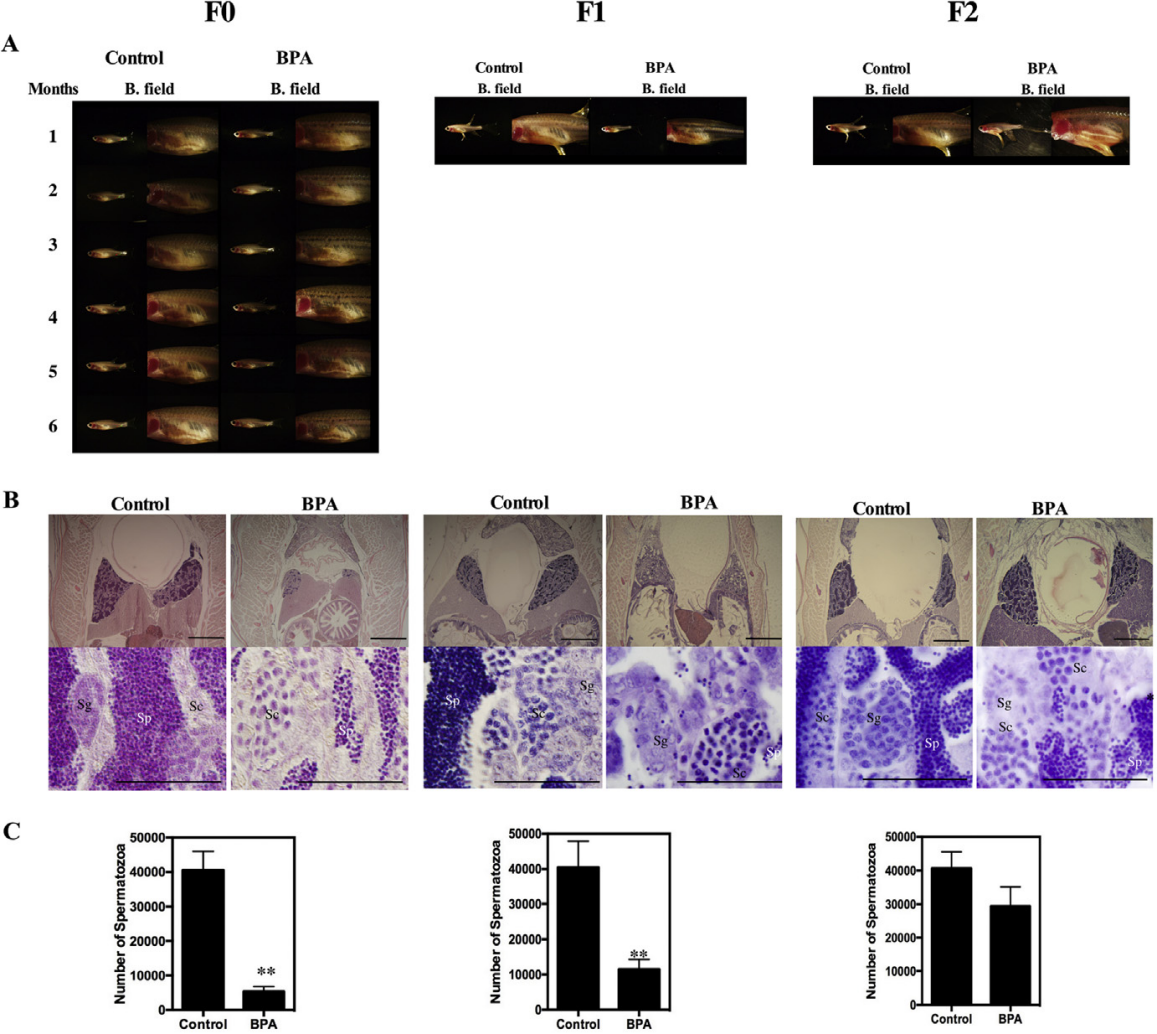
Adverse effects of BPA treatment on testes. (A) The morphology of testes during the treatment was monitored by microscopic observations performed at 1-month intervals (F0). Photographs of representative F1 and F2 males from the control (Control) and BPA-treated (BPA) groups were taken before histological analysis (F1 and F2). Photographs of whole fish and testes under bright field (B. field) are shown. (B) Sections of control (Control) and BPA-treated (BPA) males. Cysts containing spermatogonia (Sg), spermatocyte (Sc), spermatozoa (Sp) can be seen (Rupik et al., 2011). The scale bars indicate 50 μm. (C) Number of spermatozoa (Sp) in sections of control-treated (Control) and BPA-treated (BPA) males were counted in three sections prepared from three different fishes. Vertical lines indicate standard deviation. ** indicate statistically significant differences between the values for the BPA-treated group and the control group at the P < 0.01 level.

The ovaries of the BPA-treated fish gradually decreased in size and almost disappeared after 6 months of treatment (Fig. 5A). The tissues in the control and experimental groups were examined by histological observation. In the control group, the morphology of the ovarian tissues showed no significant change compared with the ovaries of untreated females (Fig. 5B). In BPA-treated fish, only previtellogenic oocytes were observed (Fig. 5B). Accordingly, the fertility of the fish was decreased (70% of the surviving fish were infertile after 4 months of treatment; Table 1). Additionally, the size of the testes and the fertility decreased in BPA-treated males (Fig. 6 and Table 1). The testes of the BPA-treated fish gradually decreased in size. Cyst structures filled with different stages of spermatozoa were observed in sections of the testes from control males (Fig. 6B). By contrast, number of cysts containing spermatozoa (Sp) was significantly decreased in BPA-treated males (Fig. 6B), thus resulting in decrease of Sp (Fig. 6C). Mating experiments were conducted to examine the fecundity of BPA-treated fish. As in females, the fertility of males was decreased (86.7% of the surviving fish were infertile; Table 1). The numbers of embryos obtained from pairings with untreated fish (BPA-treated female with normal male or normal female with BPA-treated male) were decreased, and the hatching rate of the eggs was decreased (Table 2, F1). Thus, the numbers of offspring obtained from BPA-treated fish were lower than those from control fish of both sexes. By the end of the 6 months of treatment, the rate of fertile fish was decreased to below 20% of that of the control group. Additionally, the survival rate of the offspring was significantly lower than that of the control group (Table 2, F1). These results demonstrate that changes were induced in the ovaries and testes of the zebrafish by BPA administration through dietary administration.

**Table 1:** Effects of BPA treatment on fertility of zebrafish.

			Infertile fish (%)
F0	Female	Control	10.0 (1/10)
		BPA	70.0 (7/10)
	Male	Control	20.0 (3/15)
		BPA	86.7 (13/15)
F1	Female	Control	16.0 (4/25)
		BPA	40.0 (6/15)
	Male	Control	11.1 (4/36)
		BPA	48.4 (15/31)
F2	Female	Control	22.2 (4/18)
		BPA	71.4 (15/21)
	Male	Control	13.3 (2/15)
		BPA	38.9 (7/18)

The percentages of infertile fish in each generation (F0, F1 and F2) are shown. After 4 months of treatment, pairing tests were performed three times for each individual fish. The success rates of pairings in each trial are represented as percentages. The number of fertile fish per number of tested fish is indicated in parenthesis.

**Table 2:** Effects of BPA treatment on embryonic development of zebrafish.

			Total egg no.^a^	Hatched embryo (%)^b^	Abnormal embryo (%)^c^	Survival rate (%)^d^
F1	Female	Control	94.4 ± 13.2	64.0 ± 3.7	0 (0/429)	9.1 (11/121)
		BPA	23.0 ± 5.0**	15.3 ± 3.4**	4.8 (38/798)	0.9 (7/798)
	Male	Control	100.0 ± 6.4	73.9 ± 2.7	0 (0/481)	13.9 (14/101)
		BPA	71.8 ± 10.9*	24.6 ± 4.4**	3.2 (28/862)	1.4 (12/862)
F2	Female	Control	94.4 ± 13.2	68.4 ± 6.5	0 (0/472)	30.7 (35/114)
		BPA	57.3 ± 15.5*	26.7 ± 8.1**	6.3 (24/383)	3.4 (13/383)
	Male	Control	87.5 ± 8.8	73.7 ± 5.0	0 (0/544)	36.5 (38/104)
		BPA	19.8 ± 4.8**	27.7 ± 6.4**	6.5 (27/415)	4.0 (15/415)
F3	Female	Control	104.7 ± 21.1	67.3 ± 9.1	0 (0/224)	16.8 (16/95)
		BPA	13.8 ± 7.3**	16.1 ± 6.8**	15.6 (40/257)	4.7 (12/257)
	Male	Control	90.5 ± 18.2	78.5 ± 7.4	0 (0/121)	25.8 (8/31)
		BPA	44.9 ± 15.8**	35.2 ± 8.0**	9.0 (29/321)	4.0 (13/321)

Vertical lines indicate standard deviation. ** and * indicate statistically significant differences between the values for the BPA-treated group and the control group at the P < 0.01 and P < 0.05 levels, respectively.

a The numbers of eggs obtained from the same number of control females or males (Control) and from BPA-treated females or males (BPA). The numbers from three trials were averaged.

b Percentages of hatched embryos among the obtained eggs from three trials of pairings of control females or males (Control) and BPA-treated females or males (BPA).

c Percentages of abnormal embryos from control females or males (Control) and BPA-treated females or males (BPA). The number of abnormal embryos per total number of embryos is indicated in parenthesis.

d Percentages of surviving embryos from control females or males (Control) and BPA-treated females or males (BPA). The number of surviving embryos per total number of embryos in certain set of paring is indicated in parenthesis. The data of each group from females and males beyond the F1 generations were combined as no significant changes were observed among the data.

### 3.2 Effects of BPA on the ovaries and testes of zebrafish (F1 generation)

Offspring (the F1 generation) obtained from the pairing of BPA-treated fish with untreated fish were raised in two separate groups: one group of offspring from the pairing of F0 BPA-treated females with untreated males and the other group of offspring from the pairing of F0 BPA-treated males with untreated females. From the F1 generation onward, the fish were given normal food instead of BPA-containing food. The offspring of the control fish (F1 generation) were also raised in two groups, separated based on the sex of the control parent fish, and given control food. Although most of the obviously abnormal offspring died during cultivation, the remaining juveniles matured without obvious abnormalities. However, after they became adults, abnormalities in the gonadal tissues were observed in the offspring of BPA-treated parents. Their ovaries did not fully develop, and small fluorescent oocytes appeared in almost all fish (Fig. 5A). In F1 females, as in F0 females, only previtellogenic oocytes were observed (Fig. 5B). Accordingly, the fertility of these fish was decreased (40% of the surviving fish were infertile; Table 1, F1). Additionally, abnormalities in the testes of F1 males were observed in cross sections (Fig. 6B). Number of cysts containing spermatozoa (Sp) was significantly decreased in BPA-treated males and resulted in low cell number as in F0 generation (Fig. 6B and C). As in females, the fertility of males was decreased (48.4% of the surviving fish were infertile; Table 1, F1).

The numbers of eggs obtained from pairings of F1 treated zebrafish with untreated fish were decreased compared with those of the controls, and the hatching rate of the embryos was also decreased (Table 2, F1). Thus, the number of offspring (F2 generation) obtained from BPA-treated F1 fish was significantly lower than that obtained from control F1 fish of both sexes. In addition, the survival rate of the offspring was significantly lower than that of the control offspring. These results indicate that the adverse effects of BPA were inherited to the next generation of offspring from BPA-treated parent fish due to the changes in the ovaries and testes induced by BPA administration through dietary administration.

### 3.3 Effects of BPA on the ovaries and testes of zebrafish (F2 generation)

**Fig. 7:**
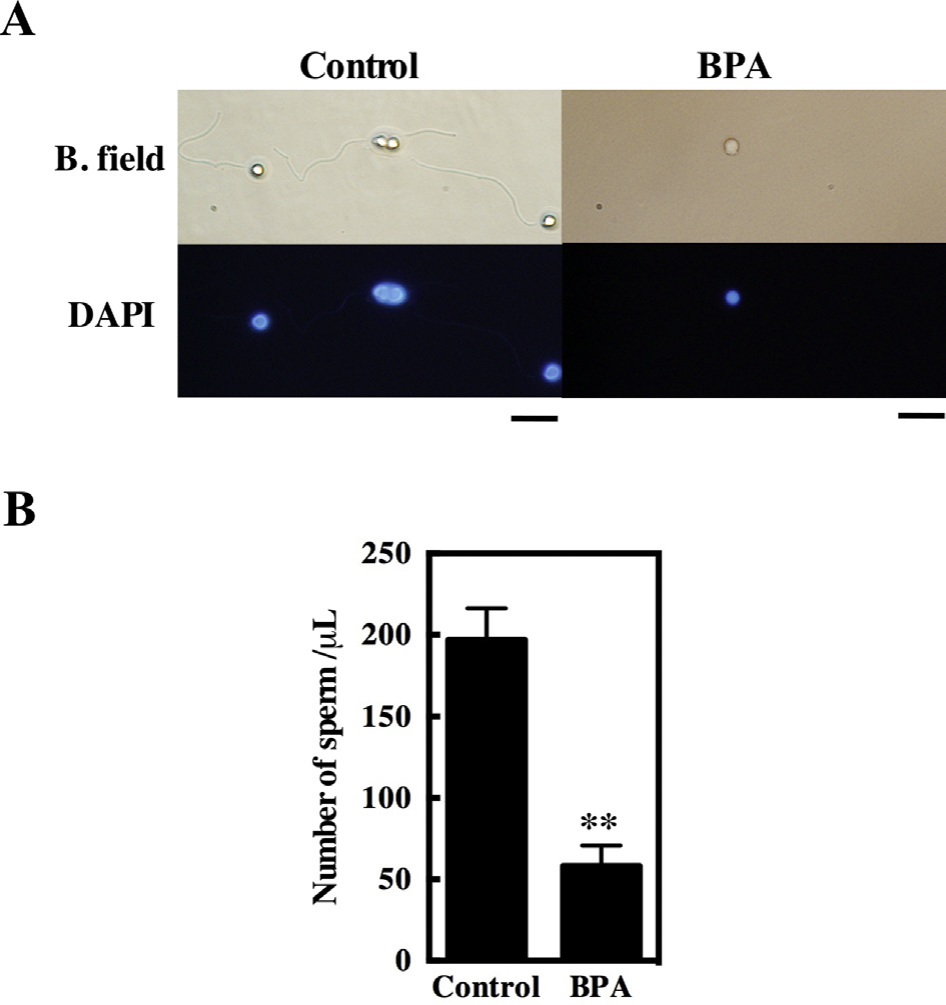
Adverse effects of BPA treatment on sperm morphology in the F2 zebrafish. (A) Sperm was collected by ejaculation and was observed after staining with DAPI. Sperm from F2 generation males from the control (Control) and BPA-treated (BPA) groups under bright field (B. field) and DAPI filter views. The scale bar indicates 10 μm. (B) The number of DAPI-positive heads of sperm or spermatozoa-like cells in 1 μl of suspensions from F2 generation males from control (Control) or BPA-treated (BPA) fish were counted (n = 3). **indicates a statistically significant difference between the values for the BPA-treated group and the control group at the P < 0.01 level.

Offspring (the F2 generation) obtained from the pairings of F1 fish with untreated fish were raised in two groups: one group from F1 fish (both females and males) that were the offspring of BPA-treated F0 females and another group from F1 fish (both females and males) that were the offspring of BPA-treated F0 males. All of these fish were given control food. The offspring of the control fish were also raised in two groups, separated based on the sex of the F0 grandparent, and given control food. Although most of the abnormal offspring died during maturation, the remaining offspring grew up without obvious abnormalities, as was the case with the F1 generation. Although no obvious abnormalities in the testes were observed in the F2 fish (Fig. 6A and B), the fertility of F2 females and males was reduced (71.4% of females and 38.9% of males were infertile; Table 1, F2). The numbers of embryos obtained from pairings with untreated fish decreased, and the hatching rate of the embryos decreased (Table 2, F3). The size of the sperm cells was similar to that observed in control males (Fig. 6B). However, when cells were extracted from the testes and the morphology of the sperm was examined by fluorescence microscopy, spermatozoa-like cells without tails were observed (Fig. 7A). The number of spermatozoa-like cells or sperm from F2 males of the BPA-treated group was significantly lower than the number of sperm from F2 males of the control group (Fig. 7B). All F2 females died after the fecundity test; therefore, histological observation was not possible.

**Fig. 8:**
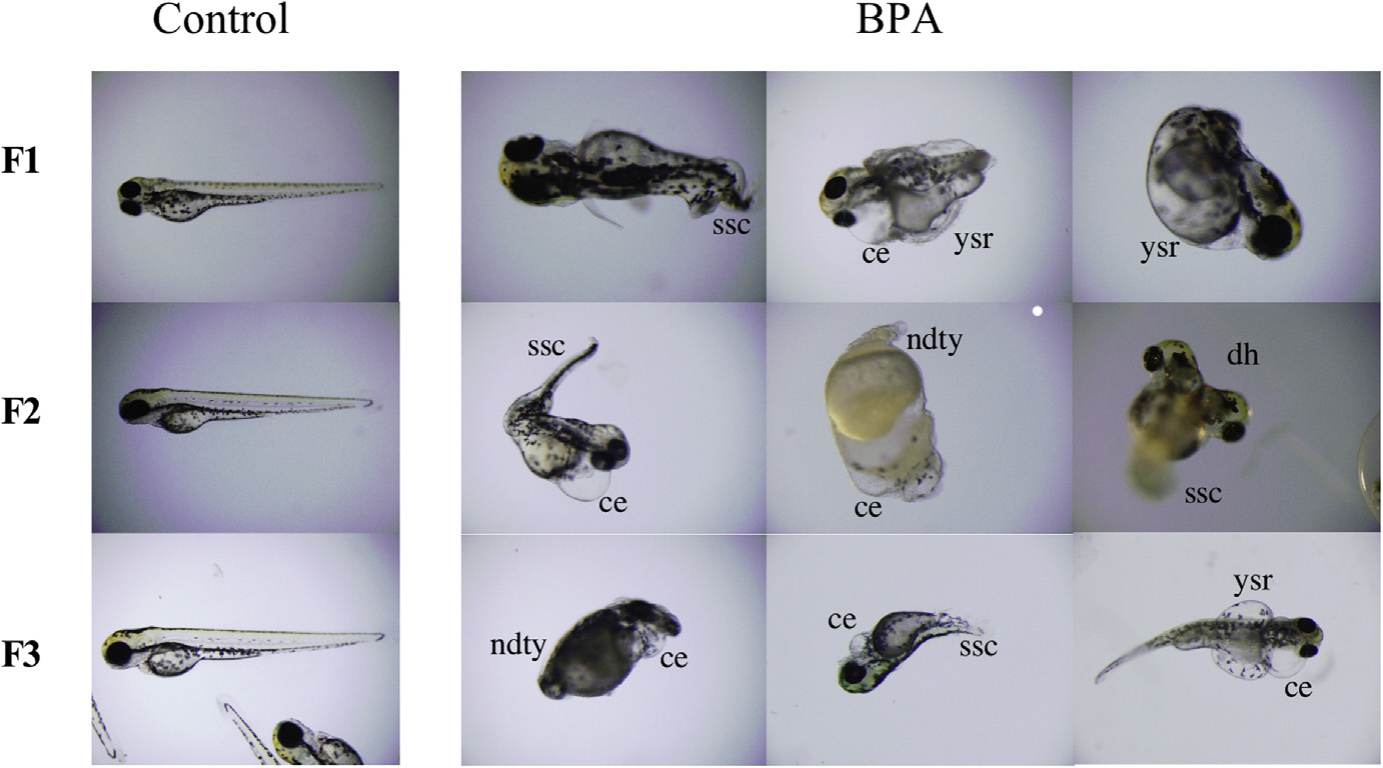
Abnormal embryonic development induced by parental BPA treatment. The morphologies of normal embryos from control (Control) fish and abnormal embryos from BPA-treated fish in generations F1 to F3. Photographs of three representative phenotypes of abnormal embryos in each generation are shown. Most of the embryos died from coagulation before hatching. Approximately five to fifteen percent of the surviving embryos exhibited abnormalities in all generations. It was observed that several abnormalities, including skeletal spine curvature (ssc), cardiac edema (ce), problems with yolk sac reabsorption (ysr), non-detachment of the tail from the yolk (dty), and double-head (dh), occurred randomly in individuals.

As shown in Fig. 8, abnormal morphological embryos developed in every generation of family lines from BPA-treated fish. A variety of types of abnormalities were found in each generation and appeared at random. By contrast, no abnormal fish developed in family lines from the control group (Fig. 8). These results indicated that the adverse BPA response is inherited in the F3 generation. Our results demonstrate the transgenerational nature of the effects of BPA in zebrafish.

## 4 Discussion

The remarkable phenomenon of the inheritance of the effects of chemicals across generations has been reported in rats (Anway et al., 2005). The mechanism of transgenerational inheritance has been found to be partially due to epigenetic modifications of the genome. Transgenerational inheritance of the effects of chemicals has also been suggested to occur in humans (Rochester, 2013). Thus, it is important that the mechanism of this inheritance be investigated in the future.

We initially observed the next-generation effect of BPA on F1 offspring during the course of sex change experiments using zebrafish (Fig. 4). In this study, we confirmed the next-generation effect and addressed the transgenerational effects of BPA. We revealed that these adverse effects are inherited through the F3 generation. In fish, in contrast to mammals, inheritance in the F2 generation corresponds to a transgenerational effect because only the oocytes and sperm appear to be affected by these chemicals in the F0 generation. Thus, we were able to evaluate the next-generation effects in the F1 generation and the transgenerational effects in the F2 generation. The toxicological effects caused in the F1 generation by the effects on oocytes and sperm is called the next-generation effect. Few but non-negligible numbers of F2 fish produced abnormal offspring. Our results demonstrate that transgenerational effects can be induced by BPA in fish and are similar to the results obtained in rats. Stage I early vitellogenic follicles became abundant in ovaries of F0 generation in BPA treated fish in this study. In zebrafish, it was shown that just 14 days treatment with BPA caused increase the percentages of atretic follicles (Molina et al., 2013). This effect thought to be induced by weak estrogenic effects of BPA and also by modification in the sex hormone balance with BPA (Mandich et al., 2007). It can be speculated that adverse effects of BPA in F0 generation were induced by these effects. Stage I follicles became abundant in ovaries of F1 generation from BPA treated fish as in F0 generation. Also, histological changes and adverse effect on testes was same between F0 and F1 generation. Thus, it seems like that conditions of gonads in F0 fish inherited by unknown mechanism to F1 fish. The most likely candidate of this mechanism is epigenetic changes in genome. Future detailed analyses of the causes of transgenerational effects should lead to a better understanding of the genetic mechanism of inheritance. Previously, transgenerational effects of BPA on heart development have been demonstrated (Lombo et al., 2015). The suggested mechanism of inheritance is the down-regulation of insulin receptors. Recently, BPA-induced changes in epigenetic patterns in zebrafish have been reported (Laing et al., 2016; Santangeli et al., 2016). A detailed analysis of the mechanism was first reported in mammals. A recent study compared the actions of a plastic compound mixture (BPA, DEHP, and DBP) with a pesticide mixture, dioxin and a hydrocarbon mixture on postnatal rats and showed that all exposure combinations induced reproductive abnormalities in F3 animals (Manikkam et al., 2013). Further detailed analysis of the effect of the plastic compound mixture was also reported (Manikkam et al., 2014). In these studies, differential DNA methylation regions (DMR) were identified on all rat chromosomes. These results suggested that a mixture of plastic-derived compounds, BPA and phthalates, can promote epigenetic transgenerational inheritance of adult onset disease. It is highly likely that the transgenerational effects of BPA are inherited as epigenetic patterns.

Transgenerational effects were reported as male-specific in many reports. However, in present study, the adverse effect of BPA was observed in both sexes. In a recent zebrafish study, adverse effects were also observed in males (Chen et al., 2015). In that study, zebrafish were treated with BPA in a group of females and males. In the present study, we separated zebrafish into groups of only females or only males to show the sex-specific effect. Females were continuously in the reproduction cycle in the mixture of females and males but the spawning cycle stopped under our experimental conditions. It can be speculated that epigenetic changes induced in oocytes remained in the ovaries of the fish in our study that resulted in transgenerational effects in females.

The cause of the next-generation effect should be addressed. A large number of F1 fish exhibited an adverse effect on their gonads, which led to low fertility. Although these fish initially appeared to be normal and healthy, closer examination revealed adverse effects on the gonads. This effect resulted in a significant decrease in the number of viable offspring. In fact, the number of hatched and surviving fish was extremely small in this generation (only 7 surviving fish from 798 eggs in the F1 from BPA-treated F0 females). Additionally, all F2 females died after they reached 6 months of age (generally zebrafish survive approximately 2 years in our laboratory). Thus, the effect should disappear gradually over time due to the death of the fish. This effect is thought to be induced by the cumulative effects of non-genomic toxic effects caused by administered chemicals, resulting in genomic effects or epigenetic changes that can be inherited. The specific origin of the adverse effects should be investigated in the future. If zebrafish incept all the given food, the amount of BPA administered to zebrafish in this study was calculated to be approximately 13.5 μg/kg body weight/day. This value is not apart form the daily exposure to human through food that is estimated at between 0.48 to 1.6 μg/kg body weight/day (Vandenberg et al., 2007). Our results emphasize the need for studies that more widely examine the transgenerational effects of endocrine disrupters.

## 5 Conclusion

In this study, we demonstrated the possibility that transgenerational inheritance of the effects of BPA can be induced in zebrafish. Specifically, we examined the effect of BPA, a well-known endocrine disruptor, on mature female and male zebrafish by adding BPA to their diet. In the females tested, BPA treatment caused ovarian retraction and reduced both fertility and the survival rate of the offspring. Similar effects were observed in treated males. The offspring of treated fish (F1 to F3 generations) demonstrated abnormalities. The mechanism of the transgenerational effect should be addressed in the future. We are also interested in investigating the underlying cause of this abnormality.

## Declarations

### Author contribution statement

Afroza Akhter: Performed the experiments; Analyzed and interpreted the data; Contributed reagents, materials, analysis tools or data; Wrote the paper.

Mostafizur Rahaman: Performed the experiments; Analyzed and interpreted the data; Contributed reagents, materials, analysis tools or data.

Yuki Murono, Ryu-to Suzuki: Performed the experiments; Analyzed and interpreted the data.

Toshinobu Tokumoto: Conceived and designed the experiments; Analyzed and interpreted the data; Wrote the paper.

### Funding statement

This work was supported by Grants-in-Aid for Scientific Research in Priority Areas from the Ministry of Education, Culture, Sports, Science and Technology of Japan.

### Competing interest statement

The authors declare no conflict of interest.

### Additional information

No additional information is available for this paper.
