# Erratum to “Next-generation and further transgenerational effects of bisphenol A on zebrafish reproductive tissues” [Heliyon 4, (9), (September 2018), Article e00788]

**DOI:** 10.1016/j.heliyon.2022.e08810

**Published:** 2022-01-23

**Authors:** Afroza Akhter, Mostafizur Rahaman, Ryu-to Suzuki, Yuki Murono, Toshinobu Tokumoto

**Affiliations:** a Integrated Bioscience Section, Graduate School of Science and Technology, National University Corporation Shizuoka University, Oya 836, Suruga-ku, Shizuoka 422-8529, Japan; b Department of Biology, Faculty of Science, National University Corporation Shizuoka University, Oya 836, Suruga-ku, Shizuoka 422-8529, Japan

In the original published version of this article, there was a unit error in the final paragraph of the Discussion section. The sentence “*If zebrafish incept all the given food, the amount of BPA administered to zebrafish in this study was calculated to be approximately 13.5 μg/kg body weight/day*” has now been corrected to *“…13.5 mg/kg body weight/day*”. The publisher apologizes for this error. In addition, the subsequent sentence “*This value is not apart form the daily exposure to human through food that is estimated at between 0.48 to 1.6 mg/kg body weight/day (Vandenberg et al., 2007)”* has been removed from the article. The authors apologize for this error. Both the HTML and PDF versions of the article have been updated to correct the errors.

## Declaration of Interests

The authors declare no conflict of interest.

